# Diagnosis and treatment of a patient with isolated spinal granulocytic sarcoma: A case report

**DOI:** 10.3892/ol.2013.1203

**Published:** 2013-02-19

**Authors:** RUO-ZHI XIAO, ZI-JIE LONG, MU-JUN XIONG, WEN-WEN WANG, DONG-JUN LIN

**Affiliations:** 1 Department of Hematology, Third Affiliated Hospital, Sun Yat-sen University, Guangzhou, P.R. China;; 2 Sun Yat-sen Institute of Hematology, Sun Yat-sen University, Guangzhou, P.R. China

**Keywords:** isolated spinal granulocytic sarcoma, diagnosis, chemotherapy, intrathecal injection

## Abstract

A previously healthy 34-year-old female presented with a 5-month history of progressive backache and weakness in the left fingers. Magnetic resonance imaging (MRI) showed soft tissue masses in the spinal canal distributed along the nerve course. The patient’s baseline laboratory data were normal. Surgical intervention was performed and histological examination identified isolated spinal granulocytic sarcoma (GS). A bone marrow biopsy also presented normal findings. However, the patient developed numbness and pain in the right lower limb two months later. Fluorodeoxyglucose (FDG)-positron emission tomography (PET) showed FDG uptake in the left trapezius muscle, cervix uteri, iliac bone, lymphadenectasis of the pelvic wall and left axillary fossa. Cerebrospinal fluid (CSF) examination allowed a diagnosis of central nervous system leukemia (CNSL). The patient underwent chemotherapy and intrathecal injection, resulting in the elimination of the residual lesion. Correct diagnosis and adequate treatment are essential to achieve optimal results in patients with isolated spinal GS.

## Introduction

Granulocytic sarcoma (GS), also referred to as myeloid sarcoma or chloroma, is a rare malignant tumor caused by the extramedullary proliferation of myeloblasts or immature myeloid cells (1–3). GS usually occurs concomitantly with or following the diagnosis of acute myeloid leukemia (AML) (2). GS may also be a symptom of a myeloproliferative disorder or leukemic transformation in myelodysplastic syndrome (4). Isolated GS has occasionally been reported to initially present in the skin, bone, pancreas, conjunctiva, gastrointestine, cervix, vagina and mediastinum. However, isolated spinal GS, particularly with the involvement of the central nervous system (CNS), is extremely rare.

The present study describes a case of isolated spinal subdural GS and a further diagnosis of CNS leukemia (CNSL) which was successfully treated with surgery, intensive chemotherapy and intrathecal injection.

## Case report

**Figure 1:**
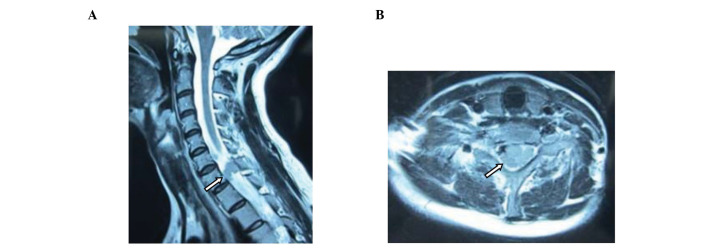
(A) MRI of the sagittal plane and (B) cross-section of the patient’s spine. T2-weighted MRI showed a large mass infiltrating the spinal canal (arrows). MRI, magnetic resonance imaging.

**Figure 2:**
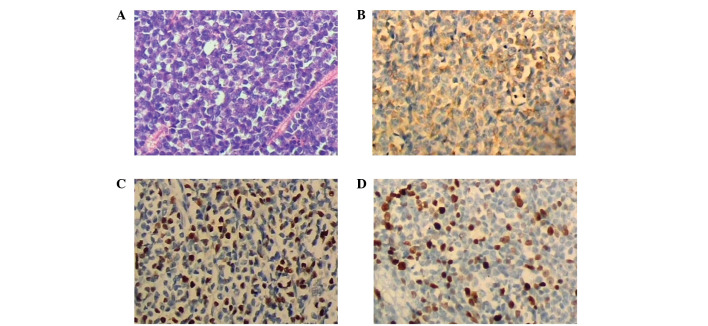
Microscopic analysis of the vertebral canal mass. (A) H&E staining. (B) Positive staining for MPO. (C) Partly positive staining for TdT. (D) Positive staining for Ki67. Magnification, ×200. HE, hematoxylin and eosin; MPO, myeloperoxidase; TdT, terminal transferase.

**Figure 3:**
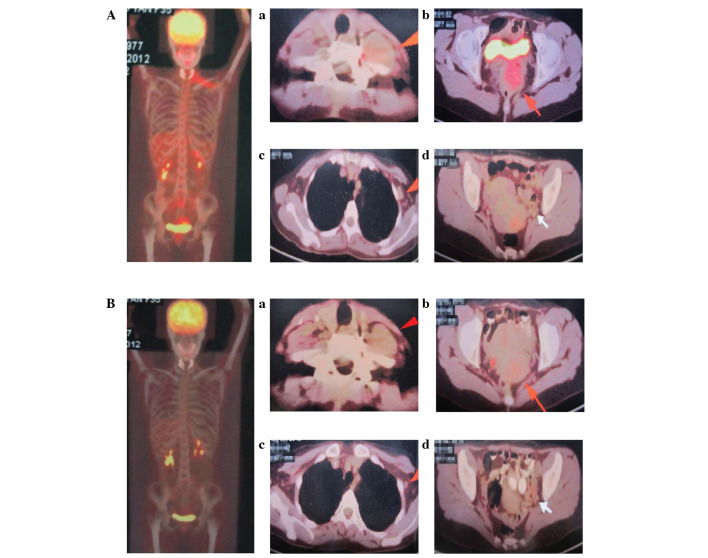
(A) FDG-PET showed hypermetabolic lesions (arrows) in the (a) left trapezius muscle, (b) cervix uteri, (c) lymphadenectasis of the left axillary fossa and (d) pelvic wall. (B) FDG-PET showed a decrease in FDG uptake following chemotherapy (a–d). FDG, fluorodeoxyglucose; PET, positron emission tomography.

A previously healthy 34-year-old female exhibited a 5-month history of progressive anesthesia and weakness in the left hand fingers. In March 2012, magnetic resonance imaging (MRI) showed that the neck and thoracic portions of the spine were involved. Soft tissue masses were observed in the spinal canal distributed along the course of the nerve root, at the C6-T1 level (Fig. 1). Blood tests showed a white blood cell count (WBC) of 6.39×10^9^/l, hemoglobin count of 119 g/l and platelet count of 200×10^9^/l. The patient immediately underwent surgical intervention with the resolution of the neurological symptoms. The pathological evaluation of the vertebral canal mass showed homogenous malignant infiltration containing round nuclei, dispersed chromatin and ill-defined eosinophilic cytoplasm (Fig. 2A). Immunohistochemical study showed the vertebral canal mass to be positive for myeloperoxidase (MPO) (Fig. 2B), partly positive for terminal transferase (TdT) (Fig. 2C), positive for Ki67 (35%, Fig. 2D) and negative for CD20, CD79a, CD138, CD15, CD3 and CD5. Bone marrow aspiration revealed a normal result. Based on these findings, the final histological diagnosis was isolated GS. The patient developed numbness and pain in the right lower limb two months later. Fluorodeoxyglucose (FDG)-positron emission tomography (PET) showed FDG uptake in the left trapezius muscle with a maximal standardized uptake value (SUV) of 2.4. The proliferation of hypermetabolic lesions was also observed in the cervix uteri, iliac bone, lymphadenectasis of the pelvic wall and left axillary fossa with maximal SUVs of 4.2, 3.0, 1.5 and 1.3, respectively (Fig. 3A). Laboratory studies revealed a hemoglobin level of 113 g/l, platelet level of 295×10^9^/l and WBC level of 9.06×10^9^/l. A bone marrow biopsy yielded a normocellular specimen. A cytogenetic study of the bone marrow cells revealed a normal karyotype. A lumbar puncture was performed and revealed elevated opening pressure (>140 mm H_2_O). Biochemical analysis of the cerebrospinal fluid (CSF) showed that the CSF WBC was 220×10^6^/l and protein was 1.19 g/l. Cytological examination of the CSF revealed a predominance of myeloid cells, including myeloblasts. The final histological diagnosis was CNSL.

Systemic induction chemotherapy was started following diagnosis and consisted of daunorubicin [90 mg/day intravenous (i.v.) on days 1, 2 and 3] and cytarabine (200 mg/day continuous i.v. on days 1–7) for 1 course, followed by pirarubicin (30 mg on day 1, 30 mg on day 2 and 40 mg on day 3) and Ara-C (200 mg/day continuous i.v. on days 1–7). During the chemotherapy, the patient also received 6 intrathecal injections containing 15 mg MTX, 50 mg Ara-C and 10 mg DXM each time. At follow-up 2 months later, the CSF WBC had disappeared and protein was 0.24 g/l. Cytological examination of the CSF did not reveal any clear myeloid tumor cells.

A visual representation of the disease site and metabolic remission was achieved by FDG-PET. The maximal SUV of the FDG uptake in the left trapezius muscle was 1.2, much lower than pre-treatment value. The maximal SUV decreased from 4.2 to 2.1 in the cervix uteri, while FDG uptake disappeared in the iliac bone, lymphadenectasis of the left axillary fossa and pelvic wall (Fig. 3B). Bone marrow examination revealed a normocellular specimen. At present, a further cycle of chemotherapy in addition to the first course is being administered.

## Discussion

GS is a localized tumor formed by primitive myeloid cells at an extramedullary site. GS was first described by Burns in 1811 and named chloroma in 1853 due to the infrequent greenish appearance observed as a result of myeloperoxydase granules in the malignant myeloid cells (5,6). GS may involve any organ system, including the skin, bone, soft tissues and lymph nodes. Spinal GS is extremely rare. It has been reported that the prevalence of GS in the spine is 1.0% among all patients with myeloid leukemia (7). GS in the absence of clinically detectable leukemia is not common and only a few cases of GS in patients without leukemia have been observed with spinal involvement (8,9). Among these, CNS involvement has been reported in 19% of non-leukemic GS patients (10).

Pathologically, the variable morphology may be misleading in GS. The correct diagnosis is sometimes challenging and is obtained in only ∼50% of non-leukemic patients due to the histological and radiological similarities to malignant lymphoma (11). The definitive diagnosis of GS requires positive immunostaining for at least 1 of the myeloid associated antigens (CD68, MPO, CD43, CD45, CD117, CD99, CD33, CD34 and CD13), as well as negative staining for the lymphoid lineages CD3 and CD20 (2,12). Bone marrow sampling is also necessary for the diagnosis of GS to assess the absence of AML. In the present case, immunohistochemical studies showed positivity for MPO and Ki67 and partly positive results for TdT, but negative results for CD20, CD79a, CD138, CD15, CD5 and CD3, indicating GS. The immunohistochemical findings were compatible with a monoblastic or myelomonoblastic variant of myeloid sarcoma. In addition, bone marrow aspiration showed a normal result, indicating no involvement of the bone marrow.

An early and precise diagnosis of spinal GS with MRI evaluation facilitates appropriate treatment with further therapy (7). However, MRI is unable to evaluate the metabolism. FDG-PET is reported to be more sensitive for the detection of malignant tumors with increased glucose metabolism (13). In the present case, FDG-PET was used to estimate the malignancy of the tumor and the treatment efficacy. It was observed that FDG-PET successfully identified the active lesion and demonstrated the malignancy. A decrease in FDG uptake was observed 2 months after treatment. The follow-up FDG-PET suggested that adequate treatment contributed to the reduction in the cellularity of the tumor.

The prognosis of patients with GS depends on the initial context in which it occurs. Out of all isolated GS patients, 66–88% develop AML within 9–11 months of diagnosis (3,14). In the present case, the patient developed CNSL 2 months after the diagnosis of GS. The optimal treatment for GS has not been fully established, partially due to the variety of clinical presentations. Chemotherapy, radiation therapy, bone marrow transplantation, surgical resection or a combination of approaches are employed in various cases. Surgery is generally reserved for patients with acute spinal cord compression or neurological symptoms. However, surgery is not always required and may worsen the prognosis due to the delayed administration of induction chemotherapy. Treating GS in the same manner as AML, even in the absence of clinically detectable leukemia has been previously reported (8). Combination treatment with radiotherapy and chemotherapy resulted in improved survival (3,10). However, isolated CNS GS and meningeal myeloid leukemia may be successfully treated without radiotherapy (16).

In accordance with the previously mentioned studies, the present patient was successfully treated using surgery and intensive anti-leukemic chemotherapy accompanied by intrathecal injections. The present case highlighted the importance of a correct diagnosis. Pre-therapeutic examinations should be the basis for the diagnosis of a mass with an atypical clinical presentation. Notably, treating GS in the same manner as AML may benefit patients with isolated spinal GS.
